# GaN-Based Ultraviolet Passive Pixel Sensor on Silicon (111) Substrate

**DOI:** 10.3390/s19051051

**Published:** 2019-03-01

**Authors:** Chang-Ju Lee, Chul-Ho Won, Jung-Hee Lee, Sung-Ho Hahm, Hongsik Park

**Affiliations:** School of Electronics Engineering, College of IT Engineering, Kyungpook National University, Daegu 41566, Korea; chjlee@knu.ac.kr (C.-J.L.); chwon@ee.knu.ac.kr (C.-H.W.); jlee@ee.knu.ac.kr (J.-H.L.)

**Keywords:** gallium nitride (GaN), ultraviolet (UV), photodetector, Schottky-barrier (SB) metal-oxide-semiconductor field-effect transistor (MOSFET), passive pixel sensor (PPS), UV image sensor

## Abstract

The fabrication of a single pixel sensor, which is a fundamental element device for the fabrication of an array-type pixel sensor, requires an integration technique of a photodetector and transistor on a wafer. In conventional GaN-based ultraviolet (UV) imaging devices, a hybrid-type integration process is typically utilized, which involves a backside substrate etching and a wafer-to-wafer bonding process. In this work, we developed a GaN-based UV passive pixel sensor (PPS) by integrating a GaN metal-semiconductor-metal (MSM) UV photodetector and a Schottky-barrier (SB) metal-oxide-semiconductor field-effect transistor (MOSFET) on an epitaxially grown GaN layer on silicon substrate. An MSM-type UV sensor had a low dark current density of 3.3 × 10^−7^ A/cm^2^ and a high UV/visible rejection ratio of 10^3^. The GaN SB-MOSFET showed a normally-off operation and exhibited a maximum drain current of 0.5 mA/mm and a maximum transconductance of 30 μS/mm with a threshold voltage of 4.5 V. The UV PPS showed good UV response and a high dark-to-photo contrast ratio of 10^3^ under irradiation of 365-nm UV. This integration technique will provide one possible way for a monolithic integration of the GaN-based optoelectronic devices.

## 1. Introduction

Ultraviolet (UV) sensors are widely used in daily life for human safety, scientific, and medical purposes, such as a fire alarm, ozone monitoring, and skin-health care [1,2,3]. To fabricate the UV photodetector, GaN is one of proper materials because it has a wide bandgap of 3.4 eV corresponding to the UV wavelength region. In the last two decades, various types of GaN-based UV photodetectors or other material-based UV photodetectors were proposed and reported in literatures [4,5,6,7,8,9,10]. The metal-semiconductor-metal (MSM)-type UV photodetector is the most appropriate device for a development of an optoelectronic integrated circuit compared to those of other-type photodetectors because of its simple fabrication process, low dark current density, and high process compatibility. To obtain more detailed information from UV light, an array-type UV pixel sensor is required. A single pixel sensor, which is a fundamental element of the image sensor, consists of a photodetector for light detection and one or more transistors for a signal transfer controller. Recently, a hybrid-type UV image sensor was reported [11]. This hybrid-type UV image sensor was fabricated by the wafer-to-wafer bonding of an AlGaN UV photodetector array and complementary metal-oxide semiconductor (CMOS) readout circuit with a backside etching process of the epitaxial substrate. To avoid the complex additional fabrication processes, such as wafer-to-wafer bonding and the backside etching of substrate, a monolithic integration technique of GaN-based UV photodetectors and transistors on a wafer should be required. Over the last two decades, GaN-based normally-off mode metal-oxide-semiconductor field-effect transistors (MOSFETs) have been intensively studied for a high-efficiency power electronic system [12,13,14,15,16,17,18,19]. In particular, the Schottky-barrier (SB)-type GaN MOSFET is the most proper device for the development of the GaN-based monolithic single pixel sensor because the device structure of the SB-MOSFET, except for the gate electrode, is the same as the MSM-type photodetector [18,19].

In this work, we demonstrated an operation of a GaN-based UV passive pixel sensor (PPS) by integrating an MSM-type UV sensor and SB-MOSFET on a highly resistive GaN layer grown on silicon substrate, which is advantageous for possible mass production. We firstly characterized the individual devices of GaN photodetector and transistor and showed the output characteristics of the fabricated GaN UV PPS under dark and 365-nm UV irradiation conditions. In addition, the effects of an asymmetric current–voltage (I–V) characteristic of GaN MSM UV photodetector on the output characteristic of PPS are discussed.

## 2. Experimental Methods

An unintentionally doped GaN layer was epitaxially grown on an n-type Si (111) substrate by using metal–organic chemical vapor deposition. To release the stress between the Si substrate and the GaN epitaxial layer, a 150-nm-thick high-temperature AlN layer and step-graded Al_x_Ga_1−x_N buffer layers (which have smaller in-plane lattice constants and thermal expansion coefficient) were grown. This can provide an additional compressive stress to suppress the tensile stress between the GaN layer and the Si substrate [19,20]. Finally, a 0.7-μm-thick crack-free and highly resistive GaN layer with a carrier concentration of lower than 2 × 10^16^ cm^−3^ was grown at 1070°C. The total growth pressure of the proposed structure was fixed at 100 Torr.

**Figure 1:**
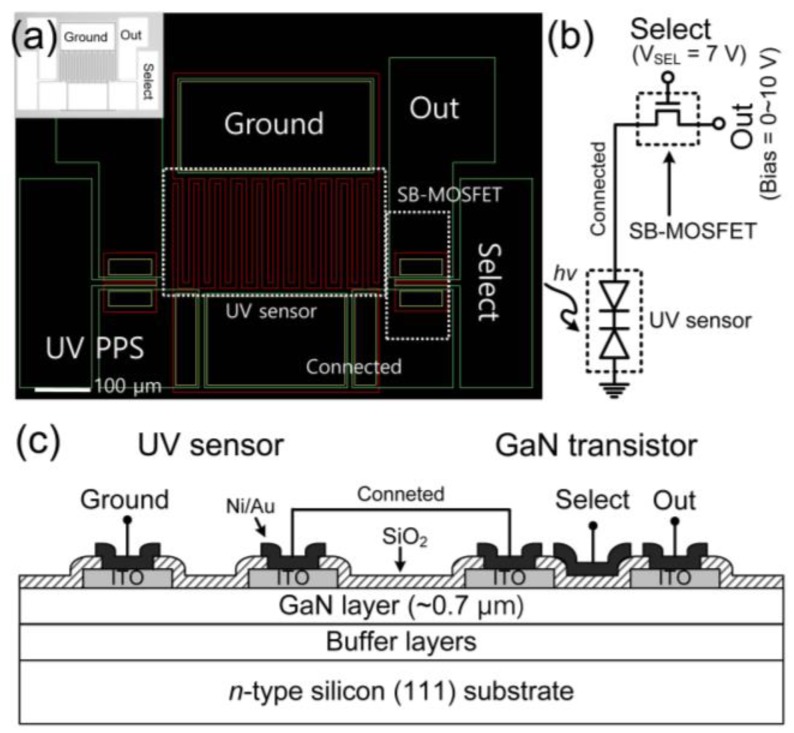
(**a**) Mask layout with pad names (inset: micro-photograph image of fabricated device), (**b**) schematic circuit diagram, and (**c**) cross-sectional view of proposed GaN ultraviolet (UV) passive pixel sensor (PPS) structure.

Figure 1a shows the mask layout of the proposed GaN UV PPS and the inset of Figure 1a shows a micro photograph image of it. The schematic circuit diagram and cross-sectional structure of the proposed GaN UV PPS are shown in Figure 1b,c. An MSM-type GaN UV photodetector, a back-to-back connection of two Schottky diodes was used for the UV-detection device, and an GaN SB-MOSFET was used for the current transfer controller. For the Schottky metal electrodes of the MSM UV sensor and SB-MOSFET, a 100-nm-thick indium-tin-oxide (ITO) was deposited by using a radio-frequency magnetron sputtering system at 300°C.

During the deposition process, we maintained the chamber pressure at 10 mTorr in Ar/O_2_ ambient (Ar:O_2_ = 1000:1). To form the gate insulator, a 25-nm-thick SiO_2_ layer was deposited by using plasma-enhanced chemical vapor deposition (PECVD) at 300°C. After the SiO_2_ layer was patterned and etched to form contact holes, Ni (20 nm) and Au (100 nm) layers were deposited for the gate electrode of SB-MOSFET and contact pad of other electrodes by using an e-beam evaporator. The area of the UV photodetector was 400 × 600 μm^2^ with a finger length of 200 μm and a finger width and space of 10 μm. The area of the SB-MOSFET was 100 × 110 μm^2^, with a gate length of 10 μm and width of 100 μm. The electrical properties of MSM UV photodetector and SB-MOSFET were characterized using the semiconductor parameter analyzer (4156C, Agilent). Photoresponsive I–V characteristics and spectral photo-responsivity characteristics were measured using a 150 W xenon arc lamp with a monochromator system (Oriel 74000, Newport) and the Newport Low-Power detector.

## 3. Results and Discussion

**Figure 2:**
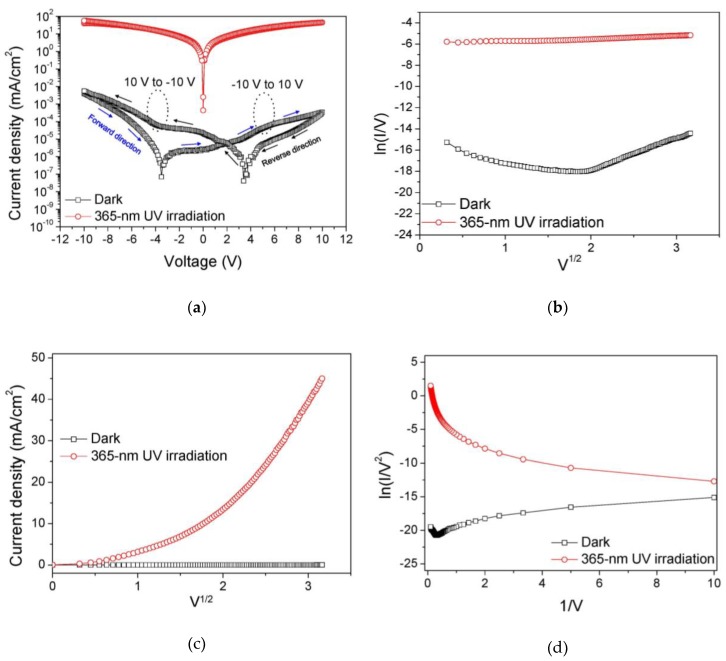
(**a**) Dark and photoresponsive I–V characteristics of the fabricated GaN metal-semiconductor-metal (MSM) photodetector under varying bias from −10 V to 10 V (forward direction) and from 10 V to −10 V (reverse direction). (**b**) Poole–Frenkel emission plot, (**c**) Schottky emission plot, and (**d**) Fowler–Nordheim tunneling plot of the I–V characteristics under dark and 365-nm UV irradiation conditions.

Figure 2a shows the dark and photoresponsive I-V characteristics of the fabricated MSM UV photodetector, where the dark current density was as low as 3.3 × 10^−7^ A/cm^2^ and photocurrent density was 4.5 × 10^−2^ A/cm^2^ at 10 V under 365-nm UV irradiation (optical power density of 447 mW/cm^2^). The low dark current value means that the Schottky contact property of ITO/GaN is good for the GaN MSM UV photodetector. The photo-to-dark contrast ratio was higher than 10^5^ at a 10 V and 10^4^ at a −10 V bias, respectively. An asymmetry of I–V characteristics and shift of current minimum in the dark state were possibly attributed to the electron trapping and de-trapping process at the ITO/GaN interface. The GaN MSM-type photodetector is effectively a back-to-back connected pair of two Schottky contacts. In the I–V measurement, the bias was swept first from −10 V to 10 V and swept back from 10 V to −10 V. As shown in Figure 2a, a significant asymmetry of I–V characteristics was observed in this measurement and the voltage values at the minimum current points of the dark I–V characteristics were not 0 V, which were shifted to −3.5 V or 3.5 V according to the direction of the bias sweep. To investigate the reason of the zero-point shift, we performed the plotting of a Poole-Frenkel emission, a Schottky emission, and a Fowler–Nordheim tunneling by using the I–V characteristics of the MSM photodetector measured under the forward bias condition. As shown in Figure 2b, the Poole-Frenkel emission is observed in the dark I–V characteristics over 4 V bias. On the other hand, the Schottky emission is not observed under dark condition as shown in Figure 2c. Moreover, not only the Poole-Frenkel emission but also a Fowler–Nordheim tunneling is clearly observed over 4 V bias, as shown in Figure 2d which means that the interface of ITO/GaN has the deep level traps and the oxide defects also exist at the interface. On the other hand, in the photoresponsive I–V characteristics under 365-nm UV irradiation, it showed an excellent bilateral symmetry without any shift of minimum current point or hysteresis, as shown in Figure 2a. The minimum current point shift in the I–V characteristic of the GaN MSM photodetector under dark could be explained by current conduction mechanisms using an electron trapping/de-trapping effect at the interface of ITO/GaN metal-semiconductor junction. In the equilibrium condition, most of the interface traps below Fermi level were occupied by electrons. If a highly negative bias was applied between ITO electrodes, the trapped electrons at the interface could be de-trapped by a trap-assisted tunneling to the GaN layer, which might contribute to a total current flow. The current is essentially the trap-assisted Poole–Frenkel emission, as clearly shown in Figure 2b. While the electrons are de-trapped, the residual unoccupied interface trap states are effectively charged positively by which the Schottky barrier height could be lowered. As the bias was changed from high voltage to low voltage in the negative bias, the band bending was reduced, and the electrons were trapped again by trap levels at ITO/GaN interface. At this condition, the electron transport mechanism consists of the thermionic emission and trapping of electrons at the interface. By the electron trapping, when the interface trap levels were occupied by electrons again, the Schottky barrier height was also recovered at the interface, which is an explanation for a positive current value of fabricated the GaN MSM Schottky diode even at the slightly negative applied bias. When comparing with Figure 2a, it is clear that the asymmetry of the I–V characteristics of the diode is caused by the interface states along ITO/GaN interface and these trap levels can be released by 365-nm UV irradiation.

**Figure 3:**
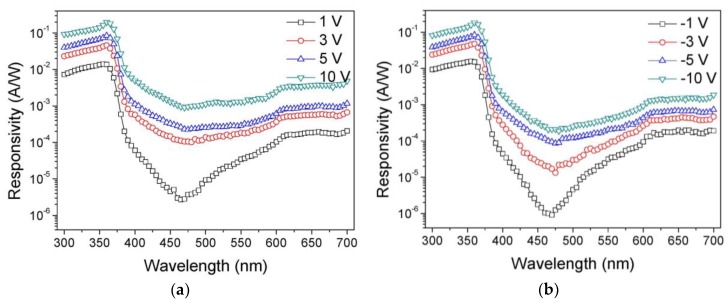
Spectral photo-responsivity characteristics of the fabricated GaN MSM UV photodetector under varying (**a**) forward and (**b**) reverse bias conditions.

Figure 3a,b show the spectral photo-responsivity characteristics of the MSM UV photodetector under varying forward and reverse bias conditions, respectively. The selectively enhanced photoresponsivity in the 350~365-nm wavelength region is attributed to the step-graded Al_x_Ga_1−x_N buffer layers [10]. The average UV-to-visible rejection ratio was about 10^2^ for any bias condition. This value is slightly lower than that of the values previously reported in the literatures [7,8,9]. The maximum value of the UV-to-visible rejection ratio was 10^3^ at 1 V bias calculated by the ratio of responsivity values at 365-nm and 450-nm wavelengths. The lowest responsivity value at the 450-nm wavelength under low bias condition means that the deep level traps corresponding to the excitation energy of 2.7 eV exist at the ITO/GaN interface. This value increases when increasing the bias, which means that the field-enhanced photo-excitation mechanism exists in the fabricated MSM photodetector. There is a photoresponse in the visible wavelength region above 600 nm as well as in the yellow band, which means that shallow level traps also exist in the ITO/GaN junction. Unlike the deep level traps, the shallow level traps were easily excited by light irradiation even at low bias condition. In the literatures, two dominant traps could exist in the epitaxially grown highly resistive GaN layer: (i) A strongly lattice-coupled deep donor and (ii) a carbon-related very deep acceptor [21,22]. These two-types of traps have the excitation energies of 1.8 and 2.85 eV, respectively, corresponding to 688 and 435 nm wavelengths, respectively. Since the excitation energy of two trap levels are closely related to the photoresponse of the GaN MSM photodetector in the visible region, we carefully conclude the undesirable visible response is attributed to the deep and shallow level traps in the ITO/GaN interface and GaN epitaxial layer. The UV-to-visible rejection ratio values are comparatively lower considering the high dark/photo contrast ratio as shown in Figure 2a, which was possibly attributed to the GaN bulk crystal defects, ITO/GaN interface defects, and SiO_2_/GaN interface defects that cause significant density of the deep level traps and shallow level traps. An asymmetry of I–V curve and photoresponse of MSM UV photodetector over the yellow band in the spectral responsivity support this analysis. But there is no bias-direction dependency in the spectral responsivity below 365 nm of wavelength.

**Figure 4:**
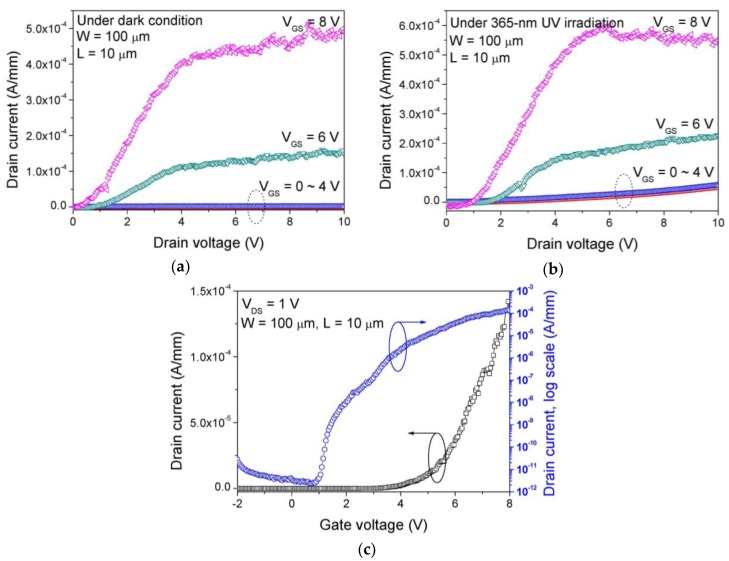
(**a**) Output I_DS_–V_DS_ characteristic under dark, (**b**) output I_DS_–V_DS_ characteristic under 365-nm UV irradiation, and (**c**) linear and log-scale transfer I_DS_–V_GS_ characteristics of the fabricated GaN Schottky-barrier (SB)-metal-oxide-semiconductor field-effect transistor (MOSFET).

**Figure 5:**
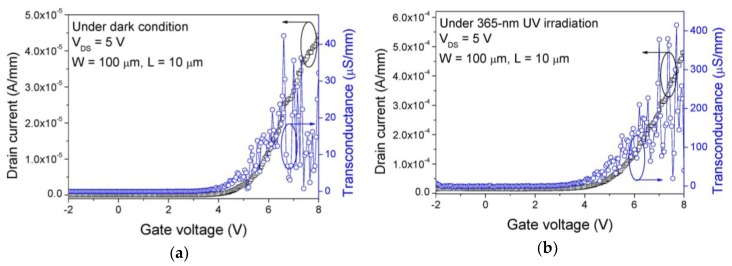
Transfer I_DS_–V_GS_ characteristics of the fabricated GaN SB-MOSFET (**a**) under dark and (**b**) under 365-nm UV irradiation.

Figure 4a shows the output I_DS_-V_DS_ characteristic of the fabricated GaN SB-MOSFET. The maximum drain current was 0.5 mA/mm at V_DS_ = 10 V. The transistor region was not entirely covered by Ni/Au for screening of UV irradiation. In order to verify that there is the UV response in the transistor, we checked the output I_DS_-V_DS_ characteristics under 365-nm UV irradiation, as shown in Figure 4b. Though the off-state drain current was only slightly enhanced by the UV irradiation, this small variation was not significant for the PPS operation because the on/off ratio of the MSM UV photodetector is higher than 10^5^ at 10 V. Figure 4c shows the linear and log-scale I_DS_–V_GS_ characteristics of the GaN SB-MOSFET in the triode region, where the threshold voltage was 4.5 V, current on/off ratio was 10^7^ at V_DS_ = 1 V. Figure 5a shows the I_DS_-V_GS_ characteristics of the GaN SB-MOSFET in the saturation region under a dark condition. The maximum transconductance was about 25 μS/mm at V_DS_ = 5 V. Except the current on/off ratio, the device performance of the fabricated GaN SB-MOSFET are relatively lower than that of values reported in literatures [18,19]. The device performance can be improved by the improvement of the crystal quality of GaN epitaxial layer and interface quality of ITO/GaN junction. The drain current and transconductance values were increased by one order of magnitude under 365-nm irradiation condition as shown in Figure 5b, which means that the photoresponsive carriers were generated at the channel region of the GaN SB-MOSFET. This undesirable photoresponsive current might also affect the off-state leakage current of the PPS as mentioned above, which can be eliminated by the layout design, for example, covering the transistor region by metal layer. In the transfer I-V characteristic, the current fluctuated according to the drain bias at a high gate bias condition, which was possibly caused by the capture and emission processes by the deep level traps at the SiO_2_/GaN interface. The SiO_2_ layer quality seemed not good enough, because it was deposited on the GaN epitaxial layer grown on silicon substrate of high lattice mismatch with GaN, which could affect the carrier transport through the GaN channel region.

**Figure 6:**
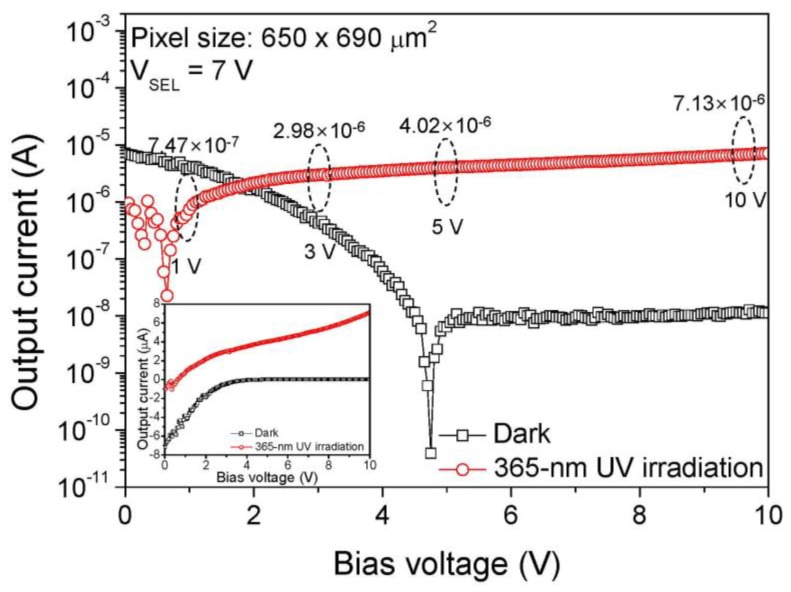
Output I–V characteristics of the fabricated GaN UV PPS with/without UV irradiation under 0–10 V bias conditions. (Inset: linear scale output I–V characteristics).

Using these two devices as a UV sensor and transistor, we developed the GaN-based UV PPS as shown in Figure 1. An MSM-type UV sensor was used for the UV-detection, and an SB-MOSFET was used for the current transfer controller. Figure 6 shows the output I-V characteristics of the fabricated GaN UV PPS with/without irradiation of 365-nm UV. The gate electrode of SB-MOSFET was used for select (SEL) electrode and drain electrode was used for output (OUT) electrode. V_SEL_ (7 V) was applied to the SEL electrode and 0–10 V bias sweep was applied to the OUT electrode. Under the dark condition, the output current changed from a high negative to a low positive value according to the increase of the bias voltages, which was closely related to the I-V characteristics of the MSM UV sensor under dark condition as shown in Figure 2a, which would be eliminated by improving the quality of the GaN epitaxial layer. In contrast, under 365-nm UV irradiation, the output current was three orders of magnitude higher than that of the dark state when the bias voltage was increased. The on/off output current ratio was 10^3^ for 365-nm UV irradiation of 447 mW/cm^2^ under 5–10 V bias. The UV on/off state is clearly distinguishable at this range of bias. The output current values under irradiation of 365-nm UV were 7.47 × 10^−7^, 2.98 × 10^−6^, 4.02 × 10^−6^, and 7.13 × 10^−6^ A at 1, 3, 5, and 10 V bias, respectively. The UV photoresponsive current of the fabricated GaN UV PPS was proportional to that of the GaN MSM UV photodetector.

## 4. Conclusions

We demonstrated the pixel operation of GaN UV PPS by integrating an MSM-type GaN UV photodetector and a GaN SB-MOSFET that was fabricated on the GaN epitaxial layer grown on silicon substrate. The fabricated GaN UV PPS showed good UV response characteristics with a high on/off ratio of 10^3^. If we improve the performance uniformity of the GaN SB-MOSFET and photoresponse characteristics of an UV photodetector, we would be able to develop the array of an active-type UV image sensor. The uniformity of the characteristics of MOSFETs fabricated on a wafer will be the key to the further integration into the GaN UV imagers.
